# Ligation of Common Carotid of Right Side and External Carotid of Left Side

**Published:** 1864-05

**Authors:** F. W. Lytle

**Affiliations:** Surgeon, 36th Ill.


ARTICLE XX.

### LIGATION OF COMMON CAROTID OF RIGHT SIDE AND EXTERNAL CAROTID OF LEFT SIDE.

Reported by F. W. LYTLE, Surgeon, 36th Ill.

Daniel Cox, æt. 28. Priv. Co. F, 15th Ind., wounded at the battle of Mission Ridge, Nov. 25th, 1863. Ball entered anterior to angle of lower maxilla on left side, fracturing the bone, making a ragged opening 1¼ inches long, passing downward under the tongue, cutting the floor of the mouth, coming out on the opposite side, a little below the cornua of the hyoid bone.

On the evening of Nov. 29th, we were called by Assistant-Surgeon Hatch, 36th Ill., to see this man, as he was bleeding profusely from wounds, and we had about one square to go to reach the building in which he was. On our arrival, found him bleeding from right side, the blood rushing from his mouth and wound in right side in a continuous stream of bright arterial blood supposed to be from a lingual artery. When we saw him, he must have lost one half gallon or nearly of blood, the bleeding having continued for several minutes, without any attempt to arrest it. The patient was placed in the semi-recumbent posture, and it was decided at once to ligate the common carotid of right side. It was utterly impossible for him to lie down; as it was, the blood flowed into his mouth with such rapidity as almost to produce strangulation.

Surgeon A. McMahon, 64th Ohio, was the operator, and from his book the following notes are taken:—

The administration of chloroform could not be entertained; and, with the assistance of Surgeon Lytle, 36th Ill., an incision was made from exit of ball down the neck, on inside of sterno-mastoid, two and a-half inches, dividing the superficial structures and deep fascia; working with handle of scalpel, succeeded in exposing the sheath with descendens noni nerve, opened the sheath; passed the aneurismal needle, armed with ligature from without, inwards, and secured the vessel. As soon as the ligature was brought home, all hemorrhage ceased.

Had we seen the patient sooner, the proper course to have pursued would have been to ligate the lingual or external carotid, but it was a question whether it would be possible to find the former at this point; and, in finding the latter, so much time would have been consumed that it would have been unnecessary to have applied the ligature. The patient would have bled to death.

Under the circumstances, ligation could only be performed at the most available point—where it would soonest arrest the hemorrhage and afford the greatest probability of saving the man's life, even at the risk of violating one of the established rules of surgery, viz., "In wounds of its deep-seated branches, ligate the external carotid." In dividing the tissues, not a single arterial or venous branch was cut, which would have rendered the operation very simple, were it not for the continued flow of blood through the wound, completely obscuring the parts; but this was remedied to a considerable extent by the valuable assistance rendered by Surgeon Lytle, in the judicious use of the sponge. The operation, to be successful, was necessarily short. The pressure applied to carotid had very little effect on the hemorrhage, as the difficulty of breathing was very great, at best, without compressing the parts about the trachea.

In tightening the ligature, I watched the patient's face to see if any effect was produced, but none was visible, except an expression of relief from the pain incidental to the operation.

During the operation, an assistant had to introduce his finger into the man's mouth to free it from the clots which interfered with respiration.

This, upon the whole, has been the most frightful case that it has been my province to witness. The blanched appearance of the face, the anxious expression of his eyes, the almost absence of pulsation at wrist, the stream of blood arching from wound as if driven by a force-pump, his shirt and bedclothes completely covered with blood,—all rendered it a sight to appal the strongest nerve. Whisky and water were freely administered during the night—with beef-tea. He rallied well, considering the vast quantity of blood he lost. Pulse small and rapid; complained of great weakness. We were fearful hemorrhage might occur from opposite side, and he was closely watched during the night.

*Nov. 30th—Morning.*—No further hemorrhage during the night. Rested tolerably well. Pulse still rapid and weak. Very much prostrated. Stimulants continued during the day. Took quite a large quantity of beef-tea, of which he was very fond.

*Evening.*—No hemorrhage. More cheerful. Has taken a good deal of nourishment. Pulse has more volume and about 100. General appearance improved.

*Dec. 1st.*—Slight hemorrhage from wound on left side, which was controlled by per sulp. ferri. *Evening.*—No return of hemorrhage.

*Dec. 2nd.*—Hemorrhage occurred again from left side this morning; appeared to come out of lower maxilla, between the ends of fractured bone, as if coming from inferior dental artery. Plugs of lint, saturated with per sulp. ferri, were inserted with temporary relief from hemorrhage.

*Evening.*—Hemorrhage again occurred from wound, the patient losing scarcely any blood during this or previous hemorrhages from this side, as he was continually under the supervision of a medical officer. This was again controlled with the iron. Finally, it was resolved that the only course to pursue, in the event of a recurrence of hemorrhage during the night, was to ligate the external carotid of left side.

About 12 o'clock, M., bleeding again occurred with considerable force, when Surgeon Herman E. Hasse, 24th Wis. Vol. (who had charge of the ward, but was not present during the previous operation), performed the operation of ligating the external carotid of left side, when the hemorrhage entirely ceased.

*Dec. 4th.*—Patient very weak. Pulse 100, small and weak. Appetite not very good. Can't take any solid food. Lives on fluids, beef-tea, farina, coffee, tea, thin gruel, whisky toddy. Milk-punch he cannot bear.

*Dec. 6th.*—General condition somewhat improved. More cheerful. Appetite better. Inclined to drowsiness. Pulsation can be felt over supra-orbital ridge, and outset of orbit on left side. No pulsation on right; face blanched.

*Dec. 9th.*—Doing well. Pulsation strong and full in temporal artery of left side; none on right. Pulse about 90, tolerably strong. Good appetite. Face lost its blanched appearance. Expressed himself doing very well.

*Dec. 11th.*—Patient improving rapidly. Pulse 86, with considerable volume. Appetite good; still takes a large quantity of beef-soup daily; thinks he will soon be able to go home. The ligature, from external carotid, separated yesterday; that from primitive, to-day. That condition of drowsiness has entirely left him. Wounds in neck granulating kindly, discharging healthy pus; no pulsation in temporal of right side.

*Dec. 16th.*—Face rather blanched; appetite not very good; complains of soreness in neck. Pulse full but somewhat gaseous. No return of drowsiness. Stimulants, with generous diet, continued.

*Dec. 20th.*—General condition improved. Pulse about 90, pretty full. Fluids pass from wound on left side, also from wound on right side. Lively and quite cheerful; piece of lower jaw rather loose; no evidence of union.

*Dec. 24th.*—Hemorrhage from small vessel—being quite superficial, controlled by iron.

From this time till the 20th January, 1864, when he was furloughed, he was carefully nourished, as before, with the addition of milk that we were enabled to procure for him. The wounds had healed, with good union of the fractured maxilla, and he was able to masticate soft food.

